# A rare case report of primary Sjögren’s syndrome with clinical characteristics similar to those of CLIPPERS

**DOI:** 10.1186/s12883-022-02945-2

**Published:** 2022-11-08

**Authors:** ChenLing Lv, FeiYan Zhu, Chao Chen, YunLing Wang, DengJun Guo, ZhenZhong Zhang

**Affiliations:** 1 Department of Neurology, Tongde Hospital of Zhejiang Province, 234 Gucui Road, 310012 Hangzhou, Zhejiang China; 2 Department of Cardiology, Hangzhou Hospital of Traditional Chinese Medicine, Hangzhou, Zhejiang China

**Keywords:** Primary Sjögren′s syndrome, CLIPPERS, Central nervous system, Magnetic resonance imaging

## Abstract

**Background:**

Primary Sjögren’s syndrome (pSS) is an autoimmune inflammatory disease characterized by dryness of the eyes, mouth and other mucous membranes. Patients with pSS can also present with extraglandular manifestations, such as pulmonary, kidney and nervous system involvement. Central nervous system (CNS) manifestations have rarely been described in pSS.

**Case presentation:**

A 33-year-old man was admitted with a one-month history of dizziness, speech disturbance, and walking instability. His brain enhanced magnetic resonance imaging (MRI) showed symmetrical, enhanced “salt-and-pepper-like” speckled lesions in the brainstem, basal ganglia, and subcortical regions, and his diagnosis was considered possible chronic lymphocytic inflammation with pontine perivascular enhancement responsive to steroids (CLIPPERS). Further examination revealed that anti-SSA antibody was positive, and the Schirmer test and labial salivary gland histopathology were abnormal, which supported the diagnosis of pSS.

**Conclusion:**

pSS is a chronic systemic autoimmune disease that involves neurological complications. This case suggests that CNS lesions of pSS can present with clinical and MRI findings similar to those of CLIPPERS.

## Background

Primary Sjögren′s syndrome (pSS) is an autoimmune disease mainly involving the exocrine and salivary glands, with a 10-60% incidence of neurologic involvement, but it is always neglected because of its atypical symptoms. The characteristic clinical symptoms are dry mouth, eyes and other parts due to reduced secretion from lacrimal and salivary glands. The neurologic manifestations of pSS are divided into peripheral neuropathies and central nervous system (CNS) involvement. CNS manifestations associated with pSS may mimic multiple sclerosis, encephalitis, cerebellar syndromes and movement disorders [1]. In Delalande S’s study, only 44% of patients had sicca symptoms as the first manifestation in pSS, and 68% of patients had CNS involvement [2]. Isabel Moreira also reported that CNS involvement was identified in 64% of patients before pSS was diagnosed [3]. Chronic lymphocytic inflammation with pontine perivascular enhancement responsive to steroids (CLIPPERS) is a CNS inflammatory syndrome predominantly affecting the brainstem, cerebellum and spinal cord [4]. We report a rare case with clinical characteristics similar to those of CLIPPERS but that was definitively diagnosed as pSS.

## Case presentation

A 33-year-old man who presented with dizziness, speech disturbance, and walking instability for more than one month was admitted to the hospital. He had a medical history of uveitis for several years, and glucocorticoids were used for a short time. On admission, his blood pressure was 110/70 mmHg. Physical examination showed slurred speech, poor adduction and abduction of both eyes, and positive results for both the Chaddock sign and positive ataxia test, whereas the remaining general and neurological examination results were generally normal. Multiple skin pigmentation could be seen in both lower limbs.

**Fig. 1:**
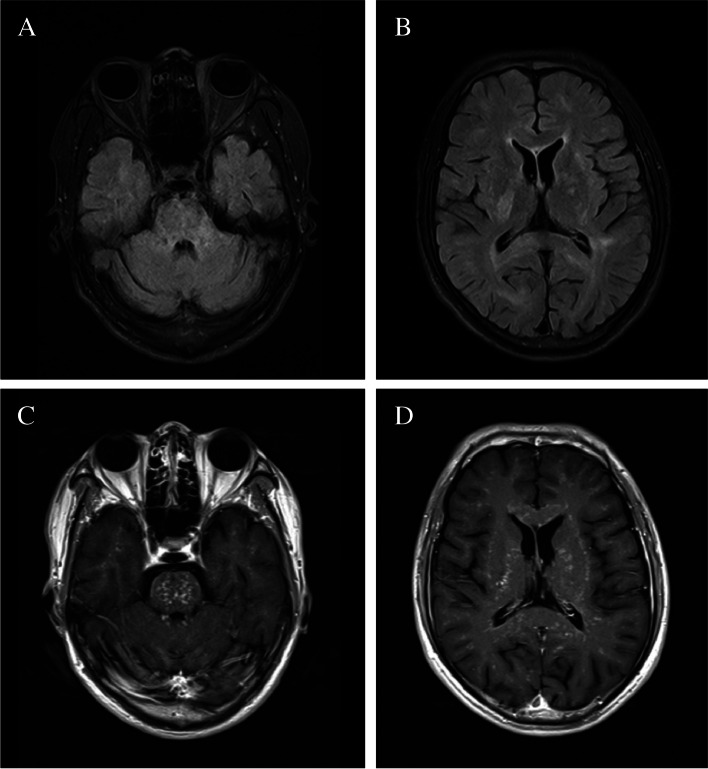
Coronal T2-weighted brain MRI demonstrates multiple lesions in the pons, basal ganglia and cerebral hemisphere with relative hyperintensity (**A** and **B**), which showed uniform enhancement with gadolinium contrast agent (**C** and **D**)

**Fig. 2:**
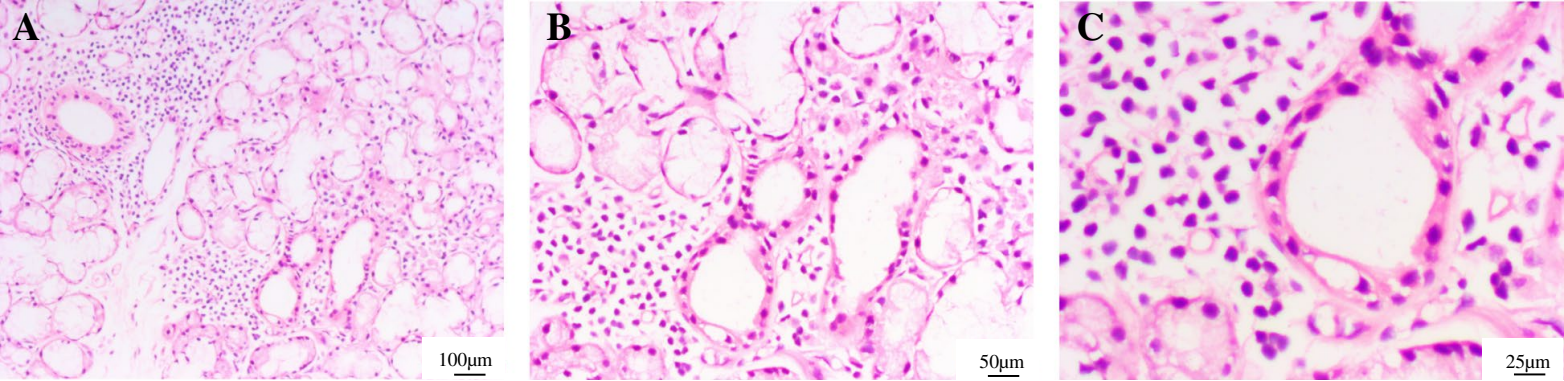
Histopathology of the labial salivary gland demonstrates a lymphocytic infiltrate around the ducts and acinus in a small area. Hematoxylin and eosin stain; **A** x100 magnification; **B** x200 magnification; **C** x400 magnification

After admission, MRI of the brain showed punctate gadolinium enhancement in the subcortical ganglia, basal ganglia and brain stem, as well as symmetrical “salt-and-pepper-like” areas (Fig. 1), and his diagnosis was considered possible CLIPPERS. Furthermore, serum anti-SSA antibody was detected in this patient. The patient’s peripheral blood cell tests showed leukopenia and thrombocytopenia, and the eosinophil ratio in the blood fraction was normal. No atypical lymphocytes were found in the peripheral blood or bone marrow smears. The value of IgG4 was 0.4 g/l, while the normal reference value was less than 2 g/l. There were no abnormalities in thyroid function, liver or kidney function, anti-HIV antibody, anti-phospholipid antibody, or anti-neutrophil cytoplasmic antibody. Cerebrospinal fluid examination showed no significant abnormalities in cell count, cerebrospinal fluid protein or CNS demyelinating antibody, including anti-AQP4. The oligoclonal band of spinal fluid was normal. In addition, chest CT showed multiple bullae in both lungs, which was considered lymphocytic interstitial pneumonia. Then the patient was evaluated with biopsy of the labial salivary gland, which revealed a lymphocytic infiltrate around the ducts and acinus in a small area (at least 50 lymphocytes/4 mm^2^), and there were no atypical lymphocytes (Fig. 2). In addition, ophthalmological involvement was assessed by the Schirmer test, which showed that 4 mm of the filter paper was moistened in 5 min, and cornea staining using fluorescein was abnormal, which supports the criteria of xerophthalmia. Based on the 2016 American College of Rheumatology/European League Against Rheumatism classification criteria for primary Sjögren’s syndrome, he was eventually diagnosed with pSS.

**Fig. 3:**
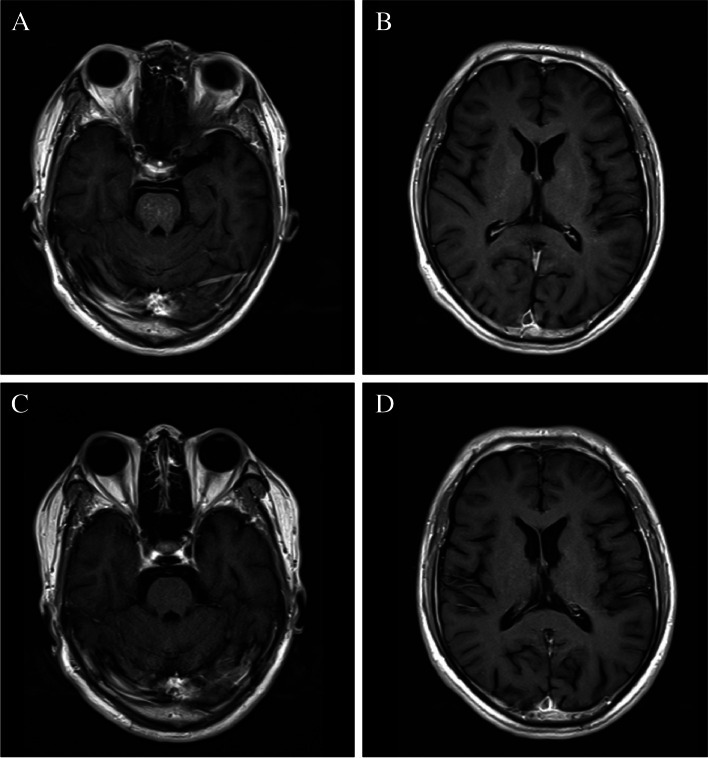
Contrast-enhanced T1-weighted MRI shows that the lesions in the pons, basal ganglia and cerebral hemisphere have been significantly reduced after 1 week of corticosteroid therapy (**A** and **B**), and follow-up cranial MRI enhancement shows that the intracranial lesions have almost disappeared after 3 months (**C** and **D**)

After discussion with the neurology and rheumatology physicians, based on anecdotal reports and clinical experience, glucocorticoid treatment was given to this patient when he was diagnosed with pSS. First, we administered methylprednisolone therapy at a dose of 1000 mg for 3 days, and then the dosage was gradually reduced to oral prednisone tablets. Due to fertility considerations, he refused to use immunosuppressants. One week later, the patient’s symptoms of slurred speech and walking instability were significantly improved. After 3 months of treatment, the above clinical symptoms improved significantly. We rechecked the contrast-enhanced MRI of the brain, and we found that the intracranial lesions had almost disappeared (Fig. 3). The patient was followed up for 1 year, and no recurrence was detected on subsequent screening.

## Discussion and conclusion

pSS is a chronic, multisystem autoimmune disorder that manifests as dryness of the eyes, mouth, and other mucous membranes, resulting from destructive mononuclear infiltration of the lacrimal and salivary glands. Sicca symptoms are absent in 47% of patients at the onset of neurologic signs, especially in those with CNS involvement [2], which demonstrates the difficulty of diagnosis and the importance of systematic screening for pSS. Morgen [5] reported that the CNS complications of pSS patients were mainly abnormal signals in the periventricular and subcortical white matter on brain MRI. In a study by Yerdelen [6], the authors found that brainstem involvement in a patient with pSS was extensive on MRI but reversible. However, the underlying pathogenesis of CNS complications in pSS remains elusive. To date, it is accepted that genetic, epigenetic and immunological alterations are responsible for its development [7]. More specifically, persistent B-cell activation and the proliferation of Th1 and Th17 cells have a pivotal role in pSS pathogenesis, thus leading to the dysregulation of the immune system and disease occurrence [8]. Nevertheless, the fact that the clinical and radiographic outcomes of patients with pSS are reversible after immunosuppressive therapy suggests that inflammation with vasogenic edema is an underlying etiology.

CLIPPERS is an inflammatory disease that was first described in 2010. The clinical manifestations of the disease are nonspecific and include gait ataxia, diplopia, sensory disturbance, and dysarthria [9]. However, the pathogenesis of CLIPPERS is unknown. Since cerebral infarction [10] and vessel abnormalities have been reported in patients with CLIPPERS [11], a vascular etiology has been proposed. Blaabjerg [12] also revealed widespread perivascular inflammation on pathology and 7 T MRI in patients with CLIPPERS. However, Tobin’s [13] study showed that none of the confirmed CLIPPERS patients had abnormal angiographic findings, and despite the presence of inflammation, there was no evidence of vasculitis on brain pathology. Clinical worsening occurred in all 12 patients with CLIPPERS when corticosteroids were discontinued. Our patient presented with clinical manifestations and radiological distribution similar to those of CLIPPERS patients. He was diagnosed with possible CLIPPERS according to the proposed diagnostic criteria [13]. However, clinical diagnosis of CLIPPERS requires the exclusion of other diseases that have alternative better explanations for clinical presentation. His clinical symptoms did not worsen after glucocorticoid withdrawal, which support that the patient did not conform to the characteristics of typical CLIPPERS. Therefore, we speculated that the cause of the patient’s symptoms was another systemic immune-mediated disease that was equally responsive to steroids.

After discussion with the pathology and hematology physicians, we did not consider the patient’s diagnosis to be lymphoma. The patient had no fever in the past month. The medical examination did not find any progressively growing lumps or lymph nodes. No atypical lymphocytes were found in the peripheral blood or bone marrow smears. HE staining of labial gland biopsy tissue showed a single focal lymphocyte infiltration rather than a diffuse sheet in the acinar, and there were no atypical lymphocytes; thus, the diagnostic criteria for non-Hodgkin lymphoma, including diffuse large B-cell lymphoma (DLBCL) and mucosa-associated lymphoid tissue (MALT) lymphoma were not met [14]. Cerebrospinal fluid examination showed no significant abnormalities in cell count or cerebrospinal fluid protein levels, indicating the absence of the central nervous system infiltrations typical of lymphoma. Moreover, chemotherapy is the main treatment for lymphoma. Glucocorticoids may be effective in the short term for patients with lymphoma, but relapse readily occurs. However, this patient received only glucocorticoids, the clinical manifestations significantly improved, and there was no recurrence after 1 year of follow-up, inconsistent with the diagnosis of typical lymphoma.

IgG4-related disease (IgG4-RD) is a multiorgan immune-mediated condition that mimics many autoimmune system diseases, such as pSS, and generally responds to glucocorticoids [15]. The patient’s IgG4 value was lower than 1.35 g/l, peripheral blood cell tests showed leukopenia and thrombocytopenia, and anti-SSA antibody and anti-Ro antibody were detected, which were not consistent with the diagnosis of IgG4-RD [16, 17].

In our case, the patient did not have the typical dry mouth and dry eye symptoms, with abnormalities in serum anti-SSA antibody and the Schirmer test. In addition, biopsy of the labial salivary gland showed at least 50 lymphocyte cells per 4 mm^2^, which fulfilled the criteria of pSS [18]. Respiratory manifestations in pSS are frequently associated with extraglandular features. Interstitial lung disease is the most serious form of lung involvement because of its association with most morbidity and early mortality [19]. Furthermore, Manuel’s [20] study showed that pulmonary involvement was a common manifestation of the nonsicca clinical spectrum of pSS, and cytopenia was also a laboratory marker at diagnosis of the systemic expression of pSS. In our case, the patient’s chest CT showed lymphocytic interstitial venereal disease, and peripheral blood tests showed cytopenia, which was consistent with the results of Manuel’s study. Autoimmunity is a possible cause of certain forms of uveitis [21], and cutaneous manifestations occur in approximately 10% of patients with pSS [22]. In our case, the patient was previously diagnosed with uveitis and responded well to glucocorticoid therapy, and there was multiple skin pigmentation in both lower limbs, which was a kind of cutaneous vasculitis. These results further support that these patients with pSS develop extraglandular manifestations, such as pulmonary, skin, hematological system and CNS involvement. Ultimately, the patient had a good prognosis with glucocorticoid therapy. Brain MRI showed that the lesion had almost disappeared after 3 months of follow-up, and no clinical symptoms recurred after more than 1 year of follow-up. Finally, the patient was diagnosed with pSS.

This is the first case report on a patient who presented clinical and MRI findings similar to those of CLIPPERS but was later definitively diagnosed with pSS. In summary, this report contributes to the clinical understanding of pSS with CNS involvement. Neurologists should be aware of the atypical clinical manifestations of pSS. However, as the pathogeneses of CLIPPERS and pSS remain unclear, further studies are needed to clarify whether there is a relationship between the two conditions.

## Data Availability

All data generated or analysed during this study are included in this published article [and its supplementary information files].

## Abbreviations

CLIPPERS: Chronic lymphocytic inflammation with pontine perivascular enhancement responsive to steroid
pSS: Primary Sjögren′s syndrome
CNS: Central nervous system
CT: Computed tomography
MRI: Magnetic resonance imaging
IgG4-RD: IgG4-related disease
